# Perception of childhood anaemia among mothers in Kumasi: a quantitative approach

**DOI:** 10.1186/s13052-018-0588-4

**Published:** 2018-11-26

**Authors:** Reindolf Anokye, Enoch Acheampong, Anthony Kwaku Edusei, Wisdom Kwadwo Mprah, Justice Ofori-Amoah, Vida Maame Kissiwaa Amoah, Vincent Ekow Arkorful

**Affiliations:** 1 Centre for Disability and Rehabilitation Studies, Department of Health Promotion, Education and Disability, Kwame Nkrumah University of Science and Technology, Kumasi, Ghana; 2 Department of Community Health, Kwame Nkrumah University of Science and Technology, Kumasi, Ghana; 3 Asokore Mampong Municipal Directorate of Health Services, Kumasi, Ghana; 4 Department of Nursing, Garden City University College, Kenyase, Ghana; 5 University for Development Studies, Tamale, Ghana

**Keywords:** Perception, Anemia, Mothers, Children under 5 years

## Abstract

**Background:**

Anaemia is the world’s second cause of disability and it affects over half of pre-school children in developing countries and at least 30–40% in industrial countries. In poorer malaria-endemic countries, anemia is one of the commonest preventable causes of death in children under 5 years. This study sought to determine the perceived causes, signs and symptoms as well prevention of childhood anaemia among mothers of children under 5 years in Kumasi, Ghana.

**Methods:**

A descriptive hospital-based cross-sectional study design with a sample of 228 patients attending the University Hospital, KNUST was used. A simple random sampling technique was applied in sampling and a structured questionnaire was used to collect data which was analyzed using SPSS statistical tools.

**Results:**

The study found that anemia was mostly perceived to be caused by poor feeding practices (43%) and fever (37%). The signs and symptoms mentioned mostly were pale conjunctiva (47%) and pale palm (44%). It was suggested that it could be prevented by giving adequate nutrition (23%), regular deworming (19%) as well as exclusive breastfeeding (25%). Mothers education and the number of children were found to be associated with the perception regarding anaemia as respondents who had completed SHS/A level were 5.14 times likely to have a higher knowledge score on Anaemia (AOR = 5.14; 95% CI; 1.01–21.8). Also, mothers who had 5 to 6 children were 1.65 times likely to have higher knowledge score on Anaemia (AOR = 1.65; 95% CI; 0.02–2.32).

**Conclusion:**

Previous experience with Anaemia and higher educational level results in better understanding of Anaemia. Therefore, extensive health education on anemia should be undertaken by the hospital authorities in collaboration with the Ministry of Health to improve knowledge of Anaemia.

## Introduction

The term Anemia denotes a reduction in the oxygen-carrying capacity of blood [1]. This occurs as a result of fewer circulating erythrocytes (red blood cells) that are needed or a decrease in the concentration of hemoglobin [1]. The World Health Organization (WHO) defines pediatric anemia according to the hemoglobin threshold in different age groups of Children [2]. Children aged 0.5 to 4.9 years with Hemoglobin threshold (gm/dl) of < 11.00; Children aged 5.0–11.9 years with Hemoglobin threshold (gm/dl) of < 11.5 and Children aged 12 to14.9 years with Hemoglobin threshold (gm/dl) of < 12.0 [2]. Anaemia is the world’s second cause of disability and it affects over half of pre-school children in developing countries and at least 30 to 40% in industrial countries [3]. In poorer malaria-endemic countries, Anaemia is one of the commonest preventable causes of death in children under 5 years [2]. More than a hundred million African children are suspected to be Anaemic [4] and this could be attributed to African cultures such as polygyny which increases family size thereby resulting in insufficient nutrient intake leading to micronutrient deficiency [5]. In Nigeria, severe anemia has been prevalent among 9.7% of children where malnutrition is 7.1% [6] and it has been ranked 5th among the ten causes of morbidity among under-five children in Ghana [7]. The nutritional survey conducted by the Ghana Health Service reported that 84% of pre-school children were Anaemic [8].

Studies on the perceptions and knowledge of anemia among mothers have provided varied results [9–14]. As such in the face of this literature lacuna, an inquisition of this sort will help generate relevant data in assessing and managing anemia whiles informing health-related policymaking. This will also facilitate the design and streamlining of novel effective and efficient interventions to address complexities and complications attributed to anemia. In addition, it will help drive health authorities to promote habits which are pertinent to ensuring the passage of sustainable strategies key to preventing anaemia.

Anemia has been ranked among the top five (5) cases of Out Patient Department (OPD) attendance and also death among children under five years at KNUST Hospital, Kumasi [15]. The consequences of the condition among “lucky to survive” children include but not limited to impaired motor development and coordination, impaired language development and poor performance in academic work as well as impaired psychological and social development. It has already been established that maternal nutritional knowledge is important as it may promote good feeding habits for the child [16]. Better understandings of how anemia is perceived will allow identification of high-risk groups and facilitate improve targeted prevention. It is on this that the current study seeks to assess the perception of anemia among mothers of children under five in Kumasi, Ghana.

## Methods

The study was carried out at the University Hospital, KNUST in the Ashanti Region of Ghana. KNUST Hospital was selected because records indicate that Anaemia ranked among the top five (5) cases of Out Patient Department (OPD) attendance and also death among children less than 5 years at the Hospital [15]. The hospital also serves densely populated surrounding communities such as Ayigya, Bomso, The KNUST community, Oforikrom, Ayeduase, Kentinkrono among others.

A quantitative approach was used whereby a descriptive hospital-based cross-sectional study design was adopted. A quantitative approach and descriptive design were used in order to classify, count, and construct statistical models in an attempt to explain the data collected. A structured questionnaire comprising of close-ended questions were used to collect data.

In selecting the respondents, a simple random sampling technique was applied. The use of a Simple random sampling technique ensured that all the mothers whose names were on the hospital’s register had an equal chance of being selected for the study. The register of mothers with children under five years attending the hospital was obtained from the Out-Patient Department (OPD). A total of 425 names were in the register and all these names were written separately on sheet of papers and placed in a container and randomly picked. Those whose names were picked were contacted and the purpose of the study was explained to them before they were included. Data were collected within a period of 2 months and 2 research assistants were employed to assist in the data collection.

The sample size was calculated using Yamane [17] formula for determining samples. For this study, 95% confidence level and Precision of 0.05 were used to calculate for the sample adopting the Equation;

$$ n=\frac{N}{1\kern0.5em +\kern0.5em N{(e)}^2} $$

Where *n* is the sample size, *N* is the population size, and *e* is the level of precision. Using 425 women with children under 5 years who had reported to the facility within the previous 3 months and applying the Yamane [17] formula, the sample size was determined.

$$ {\displaystyle \begin{array}{l}N=425\\ {}1+N{(e)}^2=1+425\kern0.24em {(.05)}^2\\ {}n=\frac{425}{\begin{array}{l}1+425\kern0.24em {(.05)}^2\\ {}n=207\end{array}}\end{array}} $$

A 10% non-respondent rate was assumed and therefore 21 were added to 207 to give us a sample size of 228. The data were analyzed using SPSS version 16.0 and presented using descriptive and inferential statistics displayed in tables.

Ethical approval was obtained from the Ethical Committee at Kwame Nkrumah University of Science and Technology, Kumasi. Verbal consent was obtained from all individual respondents included in the study.

## Results

### Demographic characteristics of respondents

Table 1 presents the Demographic Characteristics of respondents which revealed that the mean age was 31.9 ± 11.47 years and about one-third of the respondent’s (33%) were married. Traders constituted the highest proportion (29%) of the respondents and about a fourth of them (24%) had completed Junior High while about one-fifth (19%) had no form of formal education. Also, the majority (81%) of the respondents had between 1 to 4 children.

**Table 1:** showing the demographic characteristics of respondents

Variables	Characteristics	Frequency	Percentage	
Age	16–20 years	36	16%	
	21–25 years	45	20%	
	26–30 years	33	15%	
	31–35 years	55	24%	Mean = 31.9
	36 years and above	57	25%	SD = 11.47
Marital status				
	Married	76	33%	
	Single	48	21%	
	Widowed	33	15%	
	Divorced	39	17%	
	Separated	32	14%	
Ethnicity				
	Akan	109	48%	
	Ga/Adagme	37	16%	
	Ewe	43	19%	
	Gonja	39	17%	
Education				
	None	43	19%	
	Primary	42	18%	
	JHS/Middle	55	24%	
	SHS/A Level	48	21%	
	Tertiary	40	18%	
Occupation				
	Trader	65	29%	
	Farmer	28	12%	
	Unemployed	25	11%	
	Student	27	12%	
	Civil servant	35	15%	
	Artisan	48	21%	
Number of children				
	1–2 children	120	53%	
	3–4 children	64	28%	Mean = 3.5
	5–6 children	44	19%	SD =1.870

*Source:* Field survey, 2017

### Perception of Anemia

As presented in Tables 2, 27% perceived anemia as inadequate dietary intake, 62% said it is a low blood level and 11% said anemia is the loss of water from the body.

**Table 2:** Perception and Management of Anaemia

Variables	Characteristics	(*n* = 207)	%
Meaning of Anaemia			
	Inadequate dietary intake	62	27%
	Low blood level	120	62%
	Loss of water from the body	25	11%
Causes of Anaemia			
	Poor feeding practices	89	43%
	Diarrhea	9	5%
	Loss of water from the body	31	15%
	Fever	78	37%
Signs and symptoms of Anaemia			
	Pale conjunctiva	97	47%
	A runny nose	19	9%
	Pale palm	91	44%
Anemia prevention			
	Adequate nutrition	48	23%
	Resorting to spiritual help	10	5%
	Giving the child enough water	29	14%
	Regular deworming	40	19%
	Early treatment of malaria	29	14%
	Exclusive breastfeeding	52	25%
Management of Anaemia			
	Take him/her to the hospital	99	48%
	Take him/her to the herbalist	10	5%
	Give him/her locally made preparation	98	47%

*Source:* Field survey, 2017

Close to half of the respondents (43%) mentioned poor feeding practices, as the cause of Anaemia. On the signs and symptoms of Anaemia, the highest proportion of the respondents mentioned pale conjunctiva (47%). Also, almost one-fourth (23%) of the respondents were of the view that Anaemia could be prevented by giving adequate nutrition, while others resorted to spiritual help (5%), giving the child enough water (14%) as well as regular deworming (19%) with exactly one-fourth (25%), suggested that exclusive breastfeeding can prevent anaemia. Parents managed Anaemia in children mostly by taking the child to the hospital (48%) and using domestic treatment (47%) as shown in Table 2.

### Socio-demographic characteristics influencing perception of Anaemia

Table 3 shows the result of the univariate and multivariate analysis of the association between Socio-Demographic Characteristics and Perception of Anaemia. In both the univariate analysis and the multivariate analysis, Education was associated with how Anaemia was perceived. Respondents who had SHS/A level were 5.14 times likely to answer correctly to questions on Anaemia as compared to those with lower levels of education; Adjusted Odds Ratio (AOR) = 5.14 (95% confidence interval [CI]; 1.01–21.8). Respondents who had 5 to 6 children were 1.65 times likely to provide correct answers on Anaemia as compared to those with lesser number of children; Adjusted Odds Ratio (AOR) = 1.65 (95% confidence interval [CI]; 0.02–2.32).

**Table 3:** Odds ratio with 95% Confidence interval for Association between Demographic Characteristics and Knowledge of Anaemia

Variables	Anemia response		Univariate		Multivariate^a^	
	Correct (n)	Incorrect (n)	OR (95% CI)	*P*-value	AOR (95% CI)	*P*-value
Marital status						
Married	42	34	1.00	0.564	1.00	0.713
Not married	52	100	1.10 (0.89–1.97)		0.51 (0.31–7.12)	
Age						
< 20	15	21	1.00		1.00	
20 to 30	45	33	1.21 (0.17–3.84)	1.221	1.62 (0.01–4.13)	0.126
31 and above	53	59	1.18 (0.12–4.87)	1.711	2.61 (0.12–18.19)	0.239
Education						
None	35	30	1.00		1.00	
Primary	7	21	2.72 (1.89–18.1)	1.317	2.91 (0.14–6.01)	0.312
JHS/Middle	30	22	11.7 (2.11–23.01)	1.987	2.54 (1.21–7.21)	0.287
SHS/A Level	21	14	16.5 (3.54–45.49)	0.03	5.14 (1.01–21.8)	0.01
Tertiary	24	24	5.12 (0.02–5.21)	1.02	0.62 (0.02–6.00)	0.101
Number of Children						
1–2 children	73	47	1.00		1.00	
3–4 children	41	23	1.81 (0.01–4.12)	0.411	1.17 (0.12–6.01)	0.143
5–6 children	34	10	3.11(0.10–14.13)	0.010	1.65 (0.02–2.32)	0.087

^a^Mutually adjusted, *OR* Odds Ratio, *CI* Confidence Interval, *AOR* Adjusted Odds Ratio

1.00 = Reference Group

## Discussion

Children’s anemia levels are classified into three groups based on the level of hemoglobin in their blood and these are mild (hemoglobin concentration between 10.0–10.9 g/dl), moderate (hemoglobin concentration between7.0–9.9 g/dl) and severe (hemoglobin concentration less than 7.0 g/dl). The World Health Organization [2] suggests levels of hemoglobin which anemia is said to be present. These levels are hemoglobin concentration < 11 g/dL in children aged between 6 and 59 months and < 11.5 g/dL in children aged between 5 and 11 years and < 12 g/dl in older children aged between 12 and 14 years [2]. The study found that anemia is perceived as having low blood level by more than half (62%) of mothers. The mothers' description of what anaemia is could be attributed to the kind of information given to them when they visit the hospital. Anaemia was correctly perceived as caused by poor feeding practices, therefore, implying good knowledge of the cause of Anaemia. In contradiction to that, Yadav et al. [10] reported that mothers had poor knowledge regarding the cause of anemia. However, Mamta and Tamphasana [9] reported higher knowledge regarding the cause of anemia which is in line with the findings of this study. On the prevention of anemia, the majority of respondents (81%) were able to identify at least one way of preventing anemia. Some of the preventive practices of anaemia mentioned by the mothers were adequate nutrition, regular de-worming, early treatment of malaria and exclusive breastfeeding. The responses on anaemia prevention are similar to what Yadav et al. [10] reported regarding the preventive practice of anemia. Among the ways one can prevent anaemia, exclusive breastfeeding which is recommended for the first 6 months of life comes with relatively no additional cost and therefore needs to be integrated with some other types of food in order to guarantee an adequate iron intake to avoid anemia. Despite the fact that mothers knew the preventive measures of anemia when it came to the practical actions taken, 47% of them indicated that they applied domestic treatment which is not an acceptable way to manage anemia. It implies that their knowledge contradicts with their management practices on anaemia. Mothers are supposed to detect and report cases of anemia to the hospital early for appropriate treatment and interventions.

On the signs and symptoms of anemia, the majority of the respondents were of the view that pale conjunctiva and palm were visible signs and symptoms of anemia implying that respondents would easily identify anemia with those signs. The findings correspond to Adams et al. [18] who reported that over 60% of mothers recognized that an anemic child would be pale.

Determinant and contributing factors of anemia could be household characteristics such as the number of people in households, heads of households, household income, parents’ level of education, place of residence and housing facilities such as access to water and good sanitation. These could influence child care and children’s health [19–22]. According to Buor [23], there is an inverse relationship between mother’s educational level and child morbidity and mortality in Ghana where 37% of mothers of children under 5 years have never attended school, while 20% have primary education and 43% secondary or higher [14]. Anemia among children under 5 years is higher (80%) in rural areas, also higher among children of mothers with little or no education [24]. Similarly, a Kenyan study reported that a mother’s educational level is a significant socio-economic factor for the occurrence of anemia in children. A higher level of undernutrition among the under-fives was related to lower maternal education [25]. This study corroborates with the earlier studies that similarly reported a significant relationship between knowledge of anemia and education level of mothers. The current study also reported that respondents who had 5 to 6 children had a higher knowledge of Anaemia. This could be attributed to the possibility of previous experience with Anaemia which has resulted in a better understanding of it.

## Conclusion

The findings of this study suggest that anemia was mostly perceived as low blood level and caused by mainly poor feeding practices and fever. Pale conjunctiva and pale palms were the signs and symptoms usually attributed to Anaemia by the mothers. Anaemia was to be prevented by giving adequate nutrition, regular deworming as well as exclusive breastfeeding. Education and the number of children were associated with the perception regarding anemia implying that previous experience with anemia and higher educational level results to a better understanding of anemia.

## Recommendations

Authorities of the University Hospital, in discussion with the Ministry of health, government and non-governmental organisation, civil society organisations and stakeholders alike should intermittently organize outreach programmes targeting mothers who visit their hospital and those within their catchment area to improve their knowledge on anemia especially on the causes of anemia in order to equip them to detect and report suspected cases for early treatment.

The Ministry of Health, through community engagement strategies, should engage local community institutions and actors to assist grass root workers in tracking and follow up on cases and organize a periodic annual mass screening, mass de-worming, and nutrition supplementation interventions across the country.

Moreover, an extensive health education on anemia, the role of nutrition and de-worming in prevention and control should be undertaken by the hospital authorities in collaboration with not only the Ghana Health Service but also with other stakeholders.

In ensuring that, the results and output of this study translate into concrete effects as in impacting on health policy director of Ghana, we recommend a nationwide broad-based replication of this study to help address the complications, complexities, and etiology of anemia to help foster a deeper understanding of the causal factors of anemia. A study of this nature would help gather expertise knowledge whiles helping build a national consensus among health practitioners on the way forward in mounting a national strategy to fight against anemia, whiles creating national awareness among people.

There should be a shift in focus on anemia education. Strategies designed by stakeholders relative to public enlightenment should be broadened from its current state where it overly concentrates and targets mothers, to include fathers, whiles integrating extraneous, yet inextricably interlocked determinant factors such as education level, household sizes etc. In close connection with this, government pro-poor policies should be schemed to focus on under-five children from poor households and their mothers. To ensure this, the Ministry of Health, Ghana Health Service and other stakeholders like the department of social welfare, the World Health Organisation, and UNICEF could join forces to execute this proposed policy strategy.

Considering the effect of anemia on children and their motor development, especially their education, this study recommends that, public social policy interventions such as the school feeding programme should be intensified and its scope broadened to cover a host of communities, especially, ones dotted in poverty endemic areas whiles same time focusing preeminently on children under 5 years. A National Nutrition Strategy, such as one implemented by the Ministry of Health and Social Welfare in Tanzania could be replicated in Ghana.

### Limitations of the study

A limitation of the study was its focus on only a single hospital without including those attending other hospitals. The limitation affects the findings of the study in terms of its generalization to other parts of the country.

## Abbreviations

KNUST: Kwame Nkrumah University of Science and Technology
OPD: Out Patient Department
SPSS: Statistical Package for Social Sciences

## References

[1] Walsh M, Crumbie A. Watson’s clinical nursing, and related sciences E-book. NX Amsterdam: Elsevier Health Sciences; 2007.

[2] De benoist B, McLean E, Egli I, Cogswell M, World Health Organization (2008). Center for disease control and Prevention. Worldwide prevalence of anaemia 1993–2005.

[3] World Health Organization, Centers for Disease Control and Prevention (2005). Worldwide prevalence of anemia 1993–2005: WHO global database on anaemia.

[4] Davidson J (2000). Juvenile idiopathic arthritis: a clinical overview. Eur J Radiol.

[5] WHO (2000). Worldwide prevalence of anaemia 1993–2005: WHO global database on anaemia.

[6] Muoneke VU, ChidiIbekwe R (2011). Prevalence, and Aetiology of severe Anaemia in Under-5 children in Abakaliki south eastern Nigeria. PediatrTherapeut.

[7] Ghana Health Service Ghana Health Service 2007 Annual Report 2009. http://www.ghanahealthservice.org/ghs-category.php?cid=5

[8] Ghana Statistical Service (GSS), Ghana Health Services (GHS), ICF Macro (2009). Ghana Demographic and Health Survey 2008.

[9] Ojukwu JU (2002). Severe anaemia in childhood. Abstracts of proceedings: Annual general and scientific conference of Paediatric Association of Nigeria. Asaba Nig J Paediatr.

[10] Bilenko N, Yehiel M, Inbar Y, Gazala E (2007). The association between anemia in infants, and maternal knowledge and adherence to iron supplementation in southern Israel. IMAJ-RAMAT GAN.

[11] Mosleh MS (2010). Awareness of AnaemiaAmong pregnant women at UNRWA clinics in Gaza strip (doctoral dissertation, Umeå International School of Public Health).

[12] Fredanna AD, M’Cormack H, Drolet JC (2012). Assessment of Anemia knowledge, Attitudes and Behaviors Among Pregnant Women in Sierra Leone The Health Educator.

[13] Mamta DL, Devi T (2014). Prevalence of anemia and knowledge regarding anemia among reproductive-age women. IOSR J Nurs Health Sci.

[14] Yadav GV, Chakraborty A, Uechi T, Kenmochi N (2014). Ribosomal protein deficiency causes Tp53-independent erythropoiesis failure in zebrafish. Int J Biochem Cell Biol.

[15] KNUST (2015). Hospital records.

[16] Osório MM, Lira PI, Ashworth A (2004). Factors associated with Hb concentration in children aged 6-59 months in the state of Pernambuco, Brazil. Br J Nutr.

[17] Yamane T (1967). Elementary sampling theory.

[18] Adams C, Costello A, Flynn S (2009). Iron Deficiency Anemia In Ecuador: Does Education Matter.

[19] BONGAARTS JOHN (2001). Household size and composition in the developing world in the 1990s. Population Studies.

[20] Argeseanu S (2004). Risks, amenities, and child mortality in rural South Africa.

[21] Cattaneo D, Galiani S, Gertler P, Martinez R, Titiunik R (2007). Hou-sing, health, and happiness. World Bank policy research working P aper, 4214.

[22] Osório MM (2002). Determinant factors of anemia in children. J Pediatr.

[23] Buor Daniel (2003). Mothers’ education and childhood mortality in Ghana. Health Policy.

[24] Ghana Statistical Service (GSS), Ghana Health Services (GHS), ICF Macro (2004). Ghana Demographic and Health Survey 2003.

[25] Waundo J, Tuitoek PJ, Kikafunda J, Msuya J (2008). Food consumption patterns and nutrient intakes by women and under five children in the Lake Victoria Basin. African journal of environmental studies and. Development.

